# “Primary care is primary care”: Use of Normalization Process Theory to explore the implementation of primary care services for transgender individuals in Ontario

**DOI:** 10.1371/journal.pone.0215873

**Published:** 2019-04-22

**Authors:** Erin Ziegler, Ruta Valaitis, Jennifer Yost, Nancy Carter, Cathy Risdon

**Affiliations:** 1 School of Nursing, McMaster University, Hamilton, Ontario, Canada; 2 M. Louise Fitzpatrick School of Nursing, Villanova University, Villanova, Pennsylvania, United States of America; 3 Department of Family Medicine, McMaster University, Hamilton, Ontario, Canada; University of Waterloo, CANADA

## Abstract

**Background:**

In Ontario, Canada, healthcare for transgender individuals is accessed through primary care; however, there are a limited number of practitioners providing transgender care, and patients are often on waiting lists and/or traveling great distances to receive care. Understanding how primary care is implemented and delivered to transgender individuals is key to improving access and eliminating healthcare barriers. The purpose of this study is to understand how the implementation of primary care services for transgender individuals compares across various models of primary care delivery in Ontario.

**Methods:**

A qualitative, exploratory, multiple-case study guided by Normalization Process Theory (NPT) was used to compare transgender care delivery and implementation across three primary care models. Three cases known to provide transgender primary care and represent different primary care models in Ontario, Canada (i.e., family health team, community health centre, fee-for service physician) were explored. The NoMAD survey, a tool to measure implementation processes, and qualitative interviews with primary care practitioners and allied healthcare staff were administered.

**Results:**

Using the NPT framework to guide analysis, key themes emerged about successful implementation of primary care services for transgender individuals. These themes include creating a safe space for patients, identifying gaps in services, understanding practitioners’ roles, and the need for more training and education in transgender care for practitioners.

**Conclusions:**

Primary care services for transgender individuals can and should be delivered in all models of primary care. Training and awareness for healthcare practitioners are needed to develop capacity in providing primary care to transgender individuals. A greater number of practitioners and organizations are needed to take on this work, embedding and normalizing transgender care into routine practice to address barriers to access and improve quality of care for transgender individuals.

## Introduction

Canada has a publicly funded, universal health insurance system which covers all medically necessary services [1]. Yet despite this accessibility, transgender individuals continue to experience marginalization and barriers to healthcare access [2–4]. Transgender individuals have expressed anxiety over the thought of disclosing their gender identity in healthcare settings due to potential negative consequences [3, 5]. A study by Grant et al. [6] found that 28% of transgender patients had been verbally harassed and 2% had been physically assaulted while attempting to access healthcare. Transgender individuals have identified that gaining access to a practitioner who is knowledgeable about transgender healthcare is a barrier [3, 7–10]. Furthermore, organizational barriers include lack of transgender-friendly spaces and gender-neutral washrooms, binary gender documentation in electronic medical records and inappropriate reference ranges for laboratory systems [3, 4, 11]. While researchers have explored experiences of transgender individuals in obtaining care, there is a paucity of evidence about primary care provider’s experiences in the implementation and delivery of care to transgender individuals. This aim of this study is to address this gap.

Irrespective of their gender, all individuals require primary care. Primary care for transgender individuals consists of any primary healthcare services needed by individuals who identify as transgender. This can include, but is not limited to, general episodic care, chronic care management, medical supervised transition (including providing access to and monitoring of transgender hormones), and counselling. Transgender individuals have specialized healthcare needs related to their transition. Access to transgender hormones, surgery [7, 8], and knowledgeable practitioners who can provided these services has been identified as major barriers to healthcare [3, 7–10].

The size of the transgender population in Canada is based on estimates, as the current census solely collects data using male or female gender markers [12, 13]. The estimate of transgender adults in Canada is approximately 200,000, of which 77,000 live in the province of Ontario [14]. In Ontario approximately 83% of transgender individuals have a primary care physician compared to 90% of the general cisgender population [15].

In Ontario, primary care is delivered in multiple models of primary care [1, 16]. These models are generally categorized based on the remuneration method of physicians and include: Fee-for-Service (FFS), Blended Capitation, and Salaried models [1, 16, 17]. Table 1 describes the primary care models in Ontario. In the FFS model, remuneration is based on services provided [1, 18]. Family Health Organizations and Family Health Networks are blended-capitation models which allow for physicians to work in groups [17, 19, 20]. Family Health Organizations are eligible to work with a Family Health Team (FHT), an interdisciplinary team which may include nurse practitioners (NP), nurses, social workers, dietitians and pharmacists [1, 20, 21]. FHTs are part of a blended capitation or blended-salary model of remuneration [17]. Community Health Centres (CHC) are salary-based models with interdisciplinary teams providing primary care services to “hard-to-serve” communities [17, 22], such as populations with limited resources, barriers to access healthcare, or experiencing complex clinical situations. Seventy-five percent of Ontario residents and 75% of primary care physicians were enrolled in the new models of primary care, including FFS, blended capitation and salary models, by 2012 [16]. In Ontario there are 101 CHCs [23] and 184 FHTs [24].

**Table 1:** Characteristics of ontario models of primary care.

Model	Description	Renumeration	Rostered Patients	Interdisciplinary Teams
Fee-For-Service	Solo or group physicians Walk in clinics	Fee-For-Service	No	No
Family Health Organization	Group of three or more physicians	Blended Capitation	Yes	Limited
Family Health Network				
Family Health Team (FHT)	Interdisciplinary team	Blended Capitation or Blended Salary	Yes	Yes
Community Health Center (CHC)	Interdisciplinary team	Salary	No Have catchment area or defined patient populations	Yes

### Exploring implementation of primary care for transgender individuals

Understanding how primary care is implemented and delivered to transgender individuals is key to improving access and eliminating healthcare barriers. By examining implementation in different primary care models which serve transgender populations, valuable insight can be obtained on how care can be optimized in practice [25]. No primary research or conceptual literature exists on the implementation of primary care programs for the transgender population. Within the conceptual literature two programs of lesbian, gay, bisexual and transgender (LGBT) healthcare have been identified. The transgender population is often grouped together with those individuals identifying as lesbian, gay and bisexual; although not specific to the transgender population these programs may be generalizable.

Yehia et al. [26] explored the implementation of an interprofessional LGBT health program within the University of Pennsylvania. The authors developed a framework for LGBT Heath Program Development which included four steps for successful implementation: establish the need and gain support; conduct strategic planning and communication; implement activities; and finally, evaluate progress. As part of implementation, the LGBT Health Program delivered lectures on LGBT healthcare and research, hosted networking events, and advocated for LGBT awareness. The authors noted that these initiatives helped to institutionalize the program and build a foundation of support for further success.

Fenway Health in Boston Massachusetts is a leading LGBT healthcare facility that implemented a specific transgender primary care program in 2007 [27, 28]. The Fenway Health clinics have a philosophy of “accessible, patient-centered care that view gender affirmation as routine part of primary care service delivery” [27]. No information could be found specific to the implementation process, structure, or members of the Fenway Health program team. However, the key services delivered as part of the transgender healthcare program include primary care, family planning, transgender-specific healthcare, complementary and alternative medicine specialties, pharmacy, optometry, dentistry, and diagnostic imaging [27]. No research was found on how these services were implemented for the LGBT population, and more specifically there was no research that examined specific healthcare services for transgender individuals. Further research is required to understand primary care delivery to this population in order to successfully address and eliminate existing barriers and marginalization.

### Theoretical framework

The Normalization Process Theory (NPT) is a theory and conceptual framework used to understand implementation of interventions in healthcare [29]. NPT hypothesizes that practices become routine or normalized as the result of people working to enact them [30]. NPT has not been used to explore the implementation of any LGBT healthcare programs or initiatives. Within primary care, however, NPT has been used to evaluate complex mental health services [31–33], chronic kidney disease management [34], chronic back pain [35], insulin use for diabetes management [36], infertility [37], and weight management [38].

NPT identifies four theoretical constructs: coherence, cognitive participation, collective action, and reflexive monitoring [39, 40]. Coherence explores the means by which a practice is established using a set of ideas and competencies hold the practice together. Driven by the commitment of the participants, cognitive participation promotes or inhibits the legitimation of the intervention. Collective action is the material and mental work that is done to enact a practice and may include the reshaping of behaviours or actions or the reorganization of a collective purpose. In the final construct, patterns of collective action and outcomes are continuously evaluated through reflexive monitoring [39].

The purpose of this study is to understand how implementation and delivery of primary care services for transgender individuals compares across three models of primary care in Ontario. The process of implementation and specific care that is delivered in primary care to transgender individuals was explored using NPT. For the purpose of this paper the intervention explored was the delivery of primary care services for transgender individuals.

## Methods

### Case selection and boundaries of the case

A qualitative, exploratory, multiple-case study design [41] was used for this study. Three cases were purposefully selected that represented different primary care delivery models in Ontario. The comparison of programs and implementation across different primary care delivery models is vital in understanding the provision of care across Ontario. The cases invited to participate in the study included a FFS organization, a FHT and a CHC, currently providing primary care services to transgender individuals. Organizations were identified as potential sites through the primary researcher’s networks and through Rainbow Health Ontario, a province-wide program to improve access to services and to promote the health of Ontario’s LGBT communities [42]. These organizations were then invited to participate in the study.

Organizational consent was obtained first from the Director of the organization, followed by recruitment of study participants in the organizations. Primary data was collected from employees in all three cases. Individuals were eligible to participate in the study if they: 1) were currently involved in any way in the delivery of primary care services or had contact with transgender individuals in the organization; and 2) were fluent in English. Study participants included physicians, NPs, nurses (registered nurses and registered practical nurses), allied health professionals, clinical support staff (CSS) and executive directors. Allied health professionals included social workers, counsellors, psychotherapists, community support workers and program coordinators. CSS included medical receptionists and clerks. For the purpose of analysis, individuals who identified their role as either social workers, counsellors or psychotherapists were categorized under the term, “counsellor.”

### Multiple data sources and methods

Data collection included multiple sources of evidence from within each case, allowing for a broader range of data and understanding of the cases [41]. Data was collected using the NOrmalization MeAsure Development (NoMAD) tool [43], semi-structured interviews, investigator field notes and document analysis. The primary investigator recruited participants, obtained written consent to participate, and collected relevant documents. Data collection occurred in person and on site at each organization.

All participants, except clinical support staff, completed the NoMAD tool [43], a 23-item instrument based on the constructs of NPT from the viewpoint of professionals involved in the healthcare program [44]. Clinical support staff were excluded from completing the NoMAD tool as their role and activities are not directly related to the implementation and delivery of primary care services for transgender individuals. Psychometrics for the NoMAD instrument have been submitted for publication by the authors. Personal communication with T. Finch, lead author on the NoMAD manuscript, states that "The NoMAD instrument has good face validity, construct validity and internal consistency, for assessing staff perceptions of factors relevant to embedding interventions that change their work practices" [45]. The developers suggest adapting the tool for specific use by replacing the word “intervention” with a term that would be more familiar to study participants [46]. For this study, “intervention” was replaced with “primary care for transgender patients.”

Semi-structured interviews were conducted by the principal investigator using the interview guides which were developed based on the four NPT theoretical constructs [47]. One interview guide was specific for practitioners and the other guide was for CSS. Areas explored in the interviews included practitioners’ experience with transgender patients, the development and implementation of the primary care program, program demographics, and the role of the primary care team. Interviews were conducted at each clinical site and ranged in length from twenty to ninety minutes. Interviews were audiotaped and transcribed verbatim. Additionally, relevant documentary evidence including administrative reports, proposals, evaluations, and clinic forms were also collected. Data saturation was achieved through data collection.

### Analysis

Data analysis was completed by the primary investigator, who is a PhD student and primary care NP with a clinical focus in providing transgender primary care. Members of the research team (RV, NC, CR, and JY) have experience in conducting qualitative research. EZ and CR have experience in LGBT health and CR, NC and RV have experience in primary care research. Qualitative data, including participant interview and documentary evidence was analysed using NVivo 11. Analysis occurred concurrently with data collection to allow for clarification and further exploration of emerging concepts and themes in future interviews [48, 49]. Deductive codes were developed from the concepts of NPT and the research questions, and inductive coding was done as new codes emerged during the data collection [49]. Qualitative content analysis [50] and cross-case synthesis [41, 48] was completed. Once coding was completed, NVivo matrix queries were conducted to identify differences and commonalties among the cases [41, 49]. Coding and analysis were reviewed in collaboration with the research team and all members agreed on the coding structure.

Quantitative data from the NoMAD instrument was analyzed using SPSS [51]. The NoMAD instrument is divided into Option A and Option B for each question. The Likert response format in Option A was coded as 1 = *strongly disagree* to 5 = *strongly agree*. Participants were instructed to select Option B if the statement in Option A was not relevant. Option B responses include *not relevant to my role*, *not relevant at this stage* or *not relevant to the delivery of care*. Descriptive statistics were used to described participants’ responses to Option A, by NPT construct and each individual question [46]. A mean score was calculated for each NPT construct and compared across the three cases. Option B results were reported using frequencies and percentages.

The qualitative and quantitative data was analyzed separately [52]. Triangulation of the qualitative and quantitative data strengthen the findings by confirmation and corroboration of the data [49]. Convergence of qualitative and quantitative data was completed [41, 48] by examining results for each NPT construct to give an overall understanding of how primary care services for transgender individuals are implemented in Ontario. The quantitative data was used to support the qualitative data. Cross-case synthesis facilitated the comparison of commonalities and differences within the cases [41, 53].

### Validity and reliability

Yin [41] identifies four measures for quality of case study research designs: construct validity, internal validity, external validity, and reliability. The quality of this study was enhanced with the collection of multiple data sources and a chain of evidence (construct validity); pattern matching and explanation building in data analysis (internal validity); and use of replication logic in multiple case studies (external validity and reliability). Furthermore, trustworthiness [54] was ensured through multiple methods of data collection and sources (dependability); use of semi-structure interviews and fields notes (credibility); thick descriptions of the cases (transferability); and triangulation of results from interviews, documents and NoMAD tool (confirmability). Ethics Board approval was obtained from McMaster University Hamilton Integrated Research Ethics Board (HiREB #3751).

## Results

First, a brief description of each case and participants will be provided. Second, the qualitative and quantitative results will be presented within the four NPT framework constructs: coherence, cognitive participation, collective action and reflexive monitoring.

### Summary of cases

#### Case 1 –Fee-for-service

This case has been providing primary care services to transgender individuals for two years and has approximately 140 patients. One physician and one clinical support staff work for the organization located in a small-size urban community, population between 50,000–200,000 [55]. Both participated in the study.

#### Case 2 –Family health team

This case has been providing primary care services to transgender individuals for seven to ten years and has approximately 70 patients. There are twenty staff in the organization which includes physicians, NPs, allied health practitioners, clinical support staff and management. All practitioners in this organization are involved in providing primary care to transgender patients. In total, there were seven study participants including a physician (n = 1), NP (n = 1), nurse (n = 1), counsellor (n = 1), program manager (n = 1), clinical support staff (n = 1) and executive director (n = 1). The clinic is in a medium-sized urban setting, population between 200,000–500,000 [55].

#### Case 3 –Community health centre

This case serves approximately 300 transgendered patients. It is unknown how long the practice has provided care to transgender individuals. There are 45 staff in the organization. There was a total of 12 study participants including physicians (n = 2), NPs (n = 2), nurses (n = 2), counsellors (n = 3), a community support worker (n = 1), a clinical support staff (n = 1) and an executive director (n = 1). The clinic is in a metropolitan area, population between 500,000 and 1.5 million [55].

Nineteen individual interviews and 18 NoMAD surveys were completed from the three cases (Table 2). Practitioners who completed the NoMAD survey and interview included physicians (n = 4), NPs (n = 3), nurses (n = 3), counsellors (n = 4), a community support worker (n = 1) and a program manager (n = 1). Clinical support staff (n = 3) only participated in the interview as their role was not directly involved in the implementation of services. Executive directors (n = 2) only completed the NoMAD survey as their role was not involved in direct patient contact.

**Table 2:** Data collection by case.

Case	Interviews	NoMAD survey
Fee-For-Service (n = 2)	2	1
Family Health Team (n = 7)	6	6
Community Health Centre (n = 12)	11	11
TOTAL (n = 21)	19	18

Table 3 illustrates the main themes that were identified across all cases from the interviews. These results will be presented and categorized under the NPT constructs.

**Table 3:** Identified NPT Themes.

Construct	Construct Definition (39)	Major theme(s) identified
Coherence	Explores how a practice is established using a set of ideas and competencies	“It’s a safe and welcoming space”
		“Exactly that, primary care”
Cognitive Participation	The relational work that builds and sustains an intervention	Lack of access to primary care services for transgender individuals
		Understanding their individual role as practitioners
Collective Action	The operational work that people do to enact a set of practices	The need for more specific training about primary care for transgender individuals
		Guidelines and resources to improve care for transgender individuals
Reflexive Monitoring	The process of continually evaluating the outcomes	“It’s not really that difficult” to provide primary care to transgender individuals

Table 4 demonstrates the results of the NoMAD survey organized by NPT construct. Convergence of NoMAD survey results and qualitative themes is explored by construct.

**Table 4:** NoMAD results.

Item	Overall Mean (SD) (N = 18)	FFS Mean (SD) (n = 1)	FHT Mean (SD) (n = 6)	CHC Mean (SD) (n = 11)	Mann- Whitney U test (Significance)	Independent T Test (Significance)
**Coherence (Sense Making)**						
1.1-I can see how the delivery of primary care to transgender patients differs from usual primary care	3.56 (1.38)	4.00 (0.00)	3.67 (1.37)	3.45 (1.51)	-	*p =* 0.73
1.2-Staff in this organization have a shared understanding of the purpose of primary care for transgender patients	4.17 (0.86)	5.00 (0.00)	4.50 (0.55)	3.91 (0.94)	-	*p =* 0.50
1.3-I understand how the delivery of primary care to transgender patients affects the nature of my work	4.28 (0.90)	5.00 (0.00)	3.83 (1.17)	4.45 (0.69)	-	*p =* 0.21
1.4-I can see the potential value of primary care for transgender individuals for my work	4.78 (0.43)	5.00 (0.00)	4.67 (0.52)	4.82 (0.41)	-	*p =* 0.23
**Cognitive Participation**						
2.1-There are key people who drive the delivery of primary care for transgender patients forward to get others involved	4.44 (0.71)	5.00 (0.00)	4.50 (0.55)	4.36 (0.81)	*p =* 0.88	-
2.2-I believe that participating in the delivery of primary care to transgender patients is a legitimate part of my role	4.82 (0.39)	5.00 (0.00)	4.67 (0.52)	4.90 (0.32)	*p =* 0.49	-
2.3-I’m open to working with colleagues in new ways to deliver primary care to transgender patients	4.89 (0.32)	5.00 (0.00)	4.83 (0.41)	4.91 (0.30)	*p =* 0.81	-
2.4-I will continue to support the delivery of primary care services for transgender patients	4.89 (0.32)	5.00 (0.00)	5.00 (0.00)	4.82 (0.41)	*p =* 0.59	-
**Collective Action**						
3.1-I can easily integrate the delivery of transgender primary care into my existing work	4.41 (0.51)	4.00 (0.00)	4.50 (0.55)	4.40 (0.52)	-	*p =* 0.65
3.2-The delivery of transgender healthcare disrupts working relationships *	4.18 (1.02)	4.00 (0.00)	4.17 (1.60)	4.18(0.60)	-	*p =* 0.07
3.3-I have confidence in other people’s ability to deliver transgender primary care	4.11 (0.68)	4.00 (0.00)	4.17 (0.75)	4.09 (0.70)	-	*p =* 0.80
3.4-Work is assigned to those with skills appropriate to the delivery of transgender primary care	3.83 (0.71)	4.00 (0.00)	3.83 (0.41)	3.82 (0.87)	-	*p =* 0.18
3.5-Sufficient training is provided to enable staff to delivery transgender primary care	3.67 (0.84)	4.00 (0.00)	4.00 (0.63)	3.45 (0.93)	-	*p =* 0.09
3.6-Sufficient resources are available to support the delivery of transgender primary care	3.56 (1.04)	4.00 (0.00)	3.67 (1.03)	3.45 (1.13)	-	*p =* 0.52
3.7-Management adequately supports the delivery of transgender primary care	4.39 (0.85)	4.00 (0.00)	4.67 (0.52)	4.18 (0.98)	-	*p =* 0.25
**Reflexive Monitoring**						
4.1-I am aware of reports about the effects of delivery of transgender primary care	3.88 (1.05)	5.00 (0.00)	3.67 (1.03)	3.90 (1.10)	-	*p =* 0.62
4.2-The staff agree that the delivery of transgender primary care is worthwhile	4.72 (0.46)	5.00 (0.00)	4.67 (0.52)	4.73 (0.47)	-	*p =* 0.65
4.3-I value the effects that delivering transgender primary care has had on my work	4.76 (0.44)	5.00 (0.00)	4.50 (0.55)	4.90 (0.32)	-	*p =* **0.01**
4.4-Feedback about the delivery of transgender primary care can be used to improve it in the future	4.76 (0.434)	5.00 (0.00)	4.67 (0.52)	4.80 (0.42)	-	*p =* 0.31
4.5-I can modify how I delivery transgender primary care	4.47 (0.62)	5.00 (0.00)	4.40 (0.55)	4.45 (0.69)	-	*p =* 0.41

*Item reverse scored.

### Coherence

Coherence involves the “sense-making” work individuals take part in. Participants somewhat agreed that there is some difference in the delivery of primary care to transgender patients compared to non-transgender patients (*M =* 3.56, *SD =* 1.38). There was agreement on a number of items including a shared understanding of the purpose in providing primary care to transgender individuals (*M =* 4.17, *SD =* 0.86), understanding how the delivery of primary care affected their work (*M =* 4.28, *SD =* 0.90), and that they could see the potential value in their work (*M =* 4.78, *SD =* 0.43). The coherence construct was normally distributed; no statistically significant difference was identified between the CHC (*M =* 4.16, *SD =* 0.53) and FHT (*M =* 4.17, *SD =* 0.72); *t* = 0.25, *p* = 0.98. All participants selected Option A answers and therefore Option B was not selected for this construct.

Qualitative data supported the survey results for this construct. Participants agreed there was value in providing primary care to transgender individuals and viewed it the same as the primary care provided to all their patients, independent of gender. “Primary care is primary care is primary care” (CHC Nurse 1), and “I see it as exactly that, primary care” (FHT Counsellor), were common sentiments expressed throughout the cases. A physician summed it up as: “I feel like the information that you’re trying to gather isn’t any different than we try to gather from our other patients. In other words, it’s still a review of systems. It’s still a past medical history” (CHC MD 2).

All participants had a shared understanding of the value of providing primary care for transgender individuals. Providing a safe and welcoming space was seen throughout all cases as a key foundation to implementing a primary care program for transgender individuals. MD 2 described the CHC as “a positive space that we hope they [transgender individuals] would feel welcome to be able to open up about what their healthcare needs are, and not be at risk of feeling stigmatized or treated any differently than our other patients.” Key elements to providing a safe place among the cases included ensuring that the organization was inclusive and non-discriminatory, “a welcoming, inclusive place for people to access healthcare” (CHC Nurse 1). Ensuring an organization without discrimination for their patients was vital to the creation of a safe space. “There is no discrimination. There’s no anything. It’s just each person is treated as an individual” (FHT Nurse). Field observations revealed that in all cases the individual’s preferred name and pronoun were used, safe-space signage was displayed, gender-neutral washrooms were provided, and gender-neutral clinic forms were utilized.

### Cognitive participation

Cognitive participation refers to relational work done by participants as a collective to build and sustain a new intervention [39]. Participants agreed that key people drive the delivery of primary care for transgender patients forward (*M =* 4.44, *SD =* 0.71), that they were open to working with colleagues and continuing to support the delivery of transgender primary care (*M* = 4.89, *SD* = 0.32), and that the delivery of primary care to transgender patients is a legitimate part of their role (*M* = 4.82, *SD* = 0.39). The cognitive participation construct was not normally distributed; therefore, the Mann-Whitney U test was used to identify differences between the FHT and CHC. No statistically significant difference was identified between these cases, *U* = 31, *p* = 0.88. Option B, *not relevant to my role* was selected by 1 participant (5.5%) for question 2.2 which stated, “I believe that participating in the delivery of primary care to transgender patients is a legitimate part of my role.”

Qualitative data supported the survey results for this construct. All participants understood their role in delivering primary care services for their transgender patients. Most participants saw the delivery of primary care for transgender patients as being no different than care they provide to all their patients. As CHC NP 1 described, “I am providing primary care, and my role is to do that.” Additionally, many participants identified that advocacy was a key component in their role: “there’s also a role for advocacy within the centre or within the community” (CHC Counsellor 1). While all participants could define their role, many were not able to describe what other team members’ roles entailed. Many participants identified working independently and therefore may not have been aware of what others are doing for this population. However, nurses seemed to have a good understanding and were able to describe others’ roles within the CHC and FHT.

“[Patients] come in through the intake worker, and they get a file started.... From there, they go to triage by the social workers, and they have an intake with the counsellors.... And they then have a visit with the nurse first, and we do an intake where we complete their profile, including family history, their current problem list and past problem list like their past medical history, their current medications, their immunizations, and their vitals. And it’s all understood that there are no bad answers and that we’re just making sure that they’re safe to proceed to visit number two, which is a physical exam by the physician or the nurse practitioner and start in on hormones” (CHC Nurse 1).

Participants identified a lack of primary care services specifically for transgender individuals, which motivated them to provide these services within their individual roles. “Well, there’s not much out there. It’s crazy” (CHC Counsellor 2). Similar sentiments were identified in all three cases, highlighting the lack of services for transgender individuals in their respective locations. Participants identified the main factor for implementing the delivery of transgender primary care into their practice was the lack of services and unmet needs in the community. “The main thing was just that it [primary care services] wasn’t available” (FFS MD).

### Collective action

Collective action is the work that people do to enact a set of practices [39]. Participants agreed that it was easy to integrate the delivery of transgender primary care into their existing work (*M =* 4.41, *SD =* 0.51) and that management supported the care delivery (*M =* 4.39, *SD =* 0.85). Participants had confidence in other team members’ ability to deliver care (*M =* 4.11, *SD =* 0.68) and felt that work was assigned to those with the appropriate skills to deliver the primary care (*M =* 3.83, *SD =* 0.71). Participants were equivocal that enough training was provided by the organization (*M =* 3.67, *SD =* 0.84) and that sufficient resources were available (*M =* 3.56, *SD =* 1.04). Question 3.1 was reverse coded: participants agreed that the delivery of transgender care did not disrupt working relationships (*M =* 4.18, *SD =* 1.02). The collective action construct was normally distributed and no statistically significant difference was identified between the CHC (*M =* 3.60, *SD =* 0.40) and FHT (*M =* 3.81, *SD =* 0.44); *t* = 1.05, *p* = 0.31. Option B, *not relevant to my role* was selected by one participant (5.5%) for question 3.1.

Qualitative data supported the survey results for this construct. Participants in all cases identified that they received very little training about primary care for transgender individuals in their professional education. Across all practitioners’ roles, a lack of formal education was identified. Physicians in all cases identified lack of appropriate training. “Looking back at training in both medical school and residency, realised we had absolutely nothing” (FHT MD), and “I can say there was no training at all” (FFS MD). One physician, CHC MD 1 identified a brief lecture in medical school which was in the psychiatry section stating that,

“someone living in the community who was transgender actually did come and talk to us about what it was like to go through the process, which was interesting, but unfortunately there was no focus on prescribing hormones and surgery. None of the medical process was addressed at that time.”

Similarly, nurses and NPs acknowledged limited training in their respective programs. “There was some, but there was very little” (CHC NP 2) and “*I probably encountered two patients*, *but otherwise none”* (CHC Nurse 2). Counsellors also received little formal education, stating “if there was, it was quite minimal” (CHC Counsellor 1) and “I realised that I had no experience, no knowledge when it came to trans care” (FHT Counsellor). However, two counsellors did identify that they chose to explore transgender health issues further in their graduate studies. “I did my practicum with a specialist in transgendered health… that really helped me deepen my knowledge” (CHC Counsellor 3), and “I did my thesis on trans issues. I did training with [my supervisor] around writing hormone letters, and counselling with trans folks” (CHC Counsellor 2).

All participants stated that they received training from Rainbow Health Ontario on LGBTQ health and transgender primary care. While some participants obtained training independently as part of their post educational professional development, all cases provided Rainbow Health Ontario training to their staff either on-site or by supporting staff to attend training sessions. Participants in all cases also identified attending transgender healthcare conferences as a source of their continuing education.

Participants in the FHT and CHC cases identified a need for further training and education in order to deliver transgender primary care. Self identification of learning needs was evident among participants: “overall, I do still have a lot of questions about primary care in transgender individuals” (FHT MD) and “everyone’s kind of just doing what we can; kind of learning as we go. So, it would be great to have more training” (CHC Nurse 2). Furthermore, a lack of available education was identified as a barrier to addressing this learning need. “I think there needs to be more education available” (CHC Nurse 2).

All practitioners identified the Rainbow Health Ontario and Sherbourne Health Centre *Guidelines and Protocol for Hormone Therapy and Primary Health Care for Trans Clients* [56] as their primary resource used to support the delivery of primary care to transgender individuals. “I have the Sherbourne guidelines pretty much on my desk at all times” (CHC MD 2). “It’s a great assessment tool for looking at asking appropriate questions, evaluating readiness, being able to talk about gender and that exploration” (FHT Counsellor). These guidelines have also been identified as a resource that easily integrates transgender care into practice, “having the guidelines that Rainbow Health Ontario provide me makes it fairly easy” (FFS MD). Participants also identified other key resources including the Rainbow Health Ontario weekly mentorship teleconference and website and the World Professional Association for Transgender Health guidelines.

“Rainbow Health Ontario has a weekly teleconference in which providers can participate and ask questions… specific questions or general questions or reach out to the group for information or just hear what’s going on in the community in general. So, it’s another really excellent resource” (FFS MD).

Additionally, the CHC had internal resources to support practitioners providing care to transgender individuals. They developed a visit template form to guide appointments, ensuring that all the appropriate information was collected. These forms were used by physicians, NPs, nurses, and counsellors.

### Reflexive monitoring

Reflexive monitoring involves individuals’ understanding of the ways new practices affect them and those around them [39]. Participants were somewhat aware of reports about the effects of care delivery for transgender individuals (*M =* 3.88, *SD =* 1.05). They all agreed that transgender primary care was worthwhile (*M =* 4.72, *SD =* 0.46), and they value the effects it has on their work (*M =* 4.76, *SD =* 0.44). Participants also strongly agreed they could modify the way they deliver transgender primary care (*M =* 4.47, *SD =* 0.62). Overall, participants strongly agreed that they valued the effects of delivering primary care to transgender individuals had on their work (*M =* 4.76, *SD =* 0.44), however there was a statistically significant difference in the value identified by the CHC (*M =* 4.90, *SD =* 0.32) compared to the FHT (*M =* 4.50, *SD =* 0.55). The reflexive monitoring construct was normally distributed and there was no statistically significant difference was identified between the CHC (*M =* 4.53, *SD =* 0.41) and FHT (*M =* 4.38, *SD =* 0.33); *t* = -0.74, *p* = 0.44. Option B, *not relevant to my role* was selected by three individuals (16.6%) for question 4.1, 4.3 and 4.4. Option B, *not relevant at this stage* was selected by one individual (5.5%) for question 4.5 (see Table 4 for questions associated with this construct).

Qualitative data supported the Reflexive Monitoring survey results. Participants in the CHC and FHT cases acknowledged that generalized patient care surveys are distributed to all patients who attend appointments within the organization. No formal method for obtaining patient satisfaction was obtained by the FFS case; however, it was noted by both FFS participants that informal feedback has been positive. “There has been informal feedback, and it’s all been sort of good, but we haven’t looked at it in any more direct kind of way” (FFS MD).

Overall participants in all cases acknowledge that providing primary care to transgender individuals is not difficult. “I don’t think that it’s difficult at all. So, I don’t see it as anything different than any other concern that we have” (FHT NP). Physicians agreed that the medical aspects of care were not difficult and no different than providing medical care to cisgender individuals. “It’s pretty straightforward to integrate primary care and transgender care, I think, because…the medicine is pretty straightforward. It’s not really that difficult” (CHC MD 1). “The medical aspects of care are not all that complicated. They’re not outside the scope of practice of a family practitioner” (FFS MD).

## Discussion

Qualitative and quantitative data demonstrated no differences in the delivery and implementation of primary care services for transgender individuals across cases. However, while all participants valued the effects of delivering primary care to transgender individuals had on their work there was a statically significant difference between the CHC and the FHT for this item. While participants both from the CHC and the FHT strongly agreed about the value of this care to their work, the CHC (M = 4.90) was statically higher than the FHT (M = 4.50). The authors suggest this difference could be related to the organization’s mission and values. CHC’s mission is to provide care to “hard to serve”, marginalized populations and may place a higher value on delivering care to this group. Data supports that the delivery of primary care services for transgender individuals has become normalized as part of routine work within the cases.

Understanding outcomes is key to evaluating healthcare interventions; however equally important is understanding effective strategies to implement a new intervention and how well the intervention was implemented [57]. Exploring delivery of primary care for transgender individuals supported by a determinant implementation framework, such as NPT, allows for an understanding of the barriers and enablers encountered by organizations and primary care practitioners [58]. Additionally, it is important to comprehend the extent to which team members develop common practices and goals to work towards the desired outcome.

Implementation of healthcare interventions is a complex process. Using NPT facilitated understanding of how participants individually and collectively made sense of, invested in, contributed to, and evaluated the delivery of primary care services for transgender individuals. NPT served as a useful framework to guide the development of the interview questions and focus for data analysis.

The NoMAD tool was adapted for use in this study by substituting the word “intervention” with “delivery of transgender primary care.” Overall the tool was easy to administer. Currently, recommendations on the analysis of the NoMAD tool are limited; its authors suggest summarizing the frequencies of responses [43]. Further guidance on the analysis of the tool’s results, including reporting and addressing the single negative question (3.2), would strengthen the ease of use of the tool. Validity and reliability of the NoMAD tool have not been published by the developers, however outcomes were consistent with qualitative study results.

The normalization of primary care delivery for transgender individuals was present in all cases. Participants confirmed that providing primary care to transgender individuals was part of their routine, everyday practice. Results demonstrate that providing primary care was easily integrated into their work and that they valued the effect of providing this service. Transgender individuals deserve access to primary care from their primary care practitioner, but they also deserve tailored support based on their unique needs. This tailored support will require some specialized knowledge to enhance practitioners’ understanding of transgender health needs. Participants in all cases echoed that additional training and resources were needed to develop their capacity to provide primary care for this population. Although there are aspects of primary care for transgender individuals that require specialized knowledge, that does not mean that it cannot and should not be managed in primary care. As the scope of primary care practitioners has increased, they have had to develop specialized knowledge to care for a variety of patient populations with various complex healthcare needs. Some examples are addiction support [59], chronic pain in cancer survivors [60], and diabetes management [61]. Developing specialized knowledge related to transgender health is no different.

### Recommendations

Improving access to primary care services is vital to eliminate barriers to healthcare access within the transgender population. Key recommendations for implementing these services are based on the findings from the study and organized by NPT construct.

#### Coherence

Practitioners need to understand the value of providing primary care services specific to transgender individuals. The benefits to patients include improved access to care and receiving care in a safe and non-discriminatory environment. Implementing a safe space within the organization is a key responsibility of practitioners and the organization. Providing safe-space training to staff members is the first step. Formal training programs are available on making organizations positive safe spaces, such as the training module *Removing the barriers*: *Making your organization LGBT2SQ friendly* provided throughout Ontario by Rainbow Health Ontario [62]. Ensuring the organization is safe and welcoming for transgender individuals can include having gender-neutral washrooms [11], using individuals’ preferred names and pronouns, and updating clinic forms and records to remove binary gender [3, 4, 11].

#### Cognitive participation

Practitioners need to collectively define and contribute to the work and procedures needed to sustain the healthcare services provided to transgender individuals. Personal awareness of the individual practitioner roles is vital in providing primary care services. Understanding practitioners’ individual roles will highlight the strengths and limits of each role. Constant opportunity for frequent, informal shared communication is key in achieving and sustaining effective interdisciplinary collaboration and practice [63]. Ensuring practitioners are working to full scope of practice optimizes healthcare services. Additionally, by having an awareness of their different roles, practitioners can identify situations where collaboration, consultation, and referral are needed to ensure quality care is delivered by the right person at the right time. Furthermore, identifying a lack of services in the community allows practitioners to develop new services to address a gap which may be deemed a priority in the area.

#### Collective action

A key theme identified in the literature and through this study is the need for more training for practitioners. This includes training both in professional education and continuing educational opportunities for all practitioners. Medical, nursing and allied health education programs need to improve LGBT curriculum content [2, 64–66]. Providing education on general terminology, healthcare needs specific to the transgender population, and practitioners’ role in providing healthcare for this population will better prepare new practitioners for serving this community. Increased access to continuing education with LGBT content will help to increase the knowledge and skill of current practitioners. Embedding LGBT content within current programs of continuing education may increase awareness more than having specific LGBT courses [67, 68]. Embedding it in current programs may bring awareness to the concepts and highlight the need for practitioners to seek out more specific training to address their learning gaps Rainbow Health Ontario offers a variety of training workshops for practitioners to develop understanding of healthcare issues and to improve their skills in providing equitable and comprehensive services for this population [62]. Furthermore, guidelines and resources from Rainbow Health Ontario can be used to enhance current curricula.

#### Reflexive monitoring

For implementation to be assessed, formal evaluations need to be conducted. Ensuring patient satisfaction, meeting community needs, and eliminating barriers to access are key points that need to be evaluated to ensure successful implementation and continuation of service delivery. Generalized patient care surveys can be useful; however more personalized surveys to explore the healthcare needs of transgender individuals would help to develop and expand current and future programming. Additionally, building awareness within the healthcare community that primary care for transgender individuals is not difficult—it is just primary care—may help to engage and encourage practitioners to open their practices to this vulnerable population.

## Limitations

The scope of this study explored the implementation and delivery of transgender primary care as a snapshot in Ontario, Canada. A limitation of this study is that only three cases were explored and therefore results may not be generalizable to all primary care settings in Ontario. However, the purposeful selection of cases from three delivery models can help with the transferability to similar models of care delivery in other provinces and nations. Results may not be generalizable to organizations that do not have any transgender patients, or practitioners who do not have specialized knowledge of transgender health issues. Additionally, practitioners who may have been instrumental in the initial implementation may no longer be with the organization, therefore potentially limiting the understanding of implementation. This study’s cases were in urban areas of the province, therefore affecting the generalizability to northern or rural areas. The process of implementation was explored retrospectively which may limit the understanding over time and potentially have recall bias.

## Areas for future research

Further research is needed to expand the scope of the study and explore the implementation of transgender primary care services in other models of care delivery such as NP-led clinics and Aboriginal Health Centres. Furthermore, it is important to explore the prospective implementation of primary care services in organizations currently not providing service to transgender individuals. Further research on all models of primary care and delivery of services for transgender individuals is needed, both from a Canadian and international perspective. Exploring the delivery of care from the perspective of transgender individuals will improve our knowledge of factors which influence access and utilization of primary care services in this population.

## Conclusion

Using the NPT framework, we were able to explore the implementation of primary care services to transgender individuals. This study provides a window into understanding how primary care services can be implemented in Ontario for transgender individuals. Providing appropriate specialized practitioner training is key to increasing practitioners’ awareness of the transgender population’s primary care needs and buildings their capacity to provide for them. More practitioners and organizations need to embed and normalize care for transgender individuals into their routine practice to ensure this populations’ access to quality primary care services. “Primary care is primary care”—whether for the general population or transgendered individuals—is a philosophy that is within the scope of general primary care practitioners to provide.

## Supporting information

S1 File Transgender primary care—Manuscript. (DOCX)
