# Supplementary material for: “Primary care is primary care”: Use of Normalization Process Theory to explore the implementation of primary care services for transgender individuals in Ontario
Source: PLoS One. 2019 Apr 22;14(4):e0215873. doi: 10.1371/journal.pone.0215873 (PMC6476519; doi:10.1371/journal.pone.0215873)
Supplement: S1 File — (DOCX) [file pone.0215873.s001.docx]

**Abstract**

**Background:** Historically transgender individuals have experienced barriers in accessing primary care services. In Ontario, Canada, healthcare for transgender individuals is accessed through primary care, however there are a limited number of practitioners providing transgender care, and patients are often on waiting lists and/or traveling great distances to receive care. The purpose of this study is to understand how primary care is delivered and implemented in Ontario for transgender individuals.

**Methods:** A case study methodology will be used to explore this phenomenon in different primary care contexts. Normalization Process Theory is used as a guide for the study development. Three cases known to provide transgender primary care and represent different Ontario primary care models (i.e., family health team, community health centre, solo practitioner) have been identified. Comparing transgender care delivery and implementation across different models is vital to understanding how care provision to this population can be supported and spread. Qualitative interviews with primary care practitioners and clinical support staff will be conducted. Participants will also complete the NoMAD survey, a tool to measure implementation processes. The tool will be modified to explore implementation of primary care services for transgender individuals. Documentary evidence will be collected. Cross-case synthesis will be completed to compare the cases.

**Discussion:** Findings will provide an Ontario perspective on the delivery and implementation of primary care for transgender individuals in different primary care models. Barriers and facilitators in implementation and delivery will be also identified. Providing an understanding and increasing awareness of the implementation and delivery of primary care for transgender individual may help to reduce the invisibility and disparities transgender individuals experience when accessing primary care services. Understanding the delivery of primary care could allow care providers to implement primary care services for transgender individuals and improve access to healthcare for this vulnerable population.

**MeSH Keywords:** Canada; Delivery of Health Care; Primary Health Care; Transgender Persons

**Background**

The transgender community continues to represent one of the most marginalized and underserved populations in healthcare (Alegria, 2011, Bauer et al., 2009, Roberts and Fantz, 2014). Issues including discrimination, lack of provider experience and knowledge, a deficiency of services, and structural barriers contribute to the marginalization and healthcare barriers experienced by this population (Alegria, 2011, Institute of Medicine, 2011, Snelgrove et al., 2012). Language regarding gender has evolved over the years, with meanings varying over time and between disciplines (Coleman et al., 2012).Transgender describes an individual who’s gender identity or expression differs from that of their assigned sex at birth (Reisner et al., 2015, Institute of Medicine, 2011).

Estimates of the transgender population vary throughout the literature. International prevalence data over the years has ranged from 1 in 11,900 to 1 in 45,000 for male-to-female individuals and 1 in 30,400 to 1 in 200,000 for female-to-male individuals (Coleman et al., 2012). Recently, Flores (Flores et al., 2016) developed a conservative frequency estimate that 0.6% of adults in the USA identify as transgender. The most recent Canadian estimate using the estimate of 0.6% extrapolated to the 2016 Canadian census is approximately 200,000 transgender adults, with about 77,000 living in Ontario (Giblon and Bauer, 2017).

**Transgender Healthcare**

Most healthcare issues affecting transgender individuals mirror those of the general population, however transgender individuals also have unique healthcare needs and can experience distinct barriers accessing and obtaining this care (Makadon, 2011). Unique primary care needs of transgender individuals are mainly related to medically supervised transition, including providing access to and monitoring the administration and dosing of transgender hormones (Sanchez et al., 2009), which has been identified as a priority for this population (Sanchez et al., 2009, Rainbow Health Ontario, 2015, Heinz and MacFarlane, 2013).

Access to a practitioner who is knowledgeable about transgender healthcare needs has been identified as an important barrier for transgender individuals (Roberts and Fantz, 2014, Sanchez et al., 2009, Heinz and MacFarlane, 2013, Gardner and Safer, 2013, Cruz, 2014). Nurses, nurse practitioners, physicians, and other practitioners receive very little formal education about transgender health specific issues (Alegria, 2011, Roberts and Fantz, 2014). Research examining the educational preparedness of nurses and physicians in transgender knowledge is generally grouped under the broader umbrella of lesbian, gay, bisexual and transgender (LGBT) health. For the purpose of transgender competent healthcare, this is problematic as this grouping is too broad and groups together sexual preference with gender identity. White, et al. (White et al., 2015) found that medical schools in the United States and Canada teach a median of five hours of LGBT content in their required curricula. Obedine-Maliver, et al. (Obedin-Maliver et al., 2011) found that one-third of medical schools provided education on transgender hormones and or surgical transition. A study by Lim, et al. (Lim et al., 2015) found that the median time devoted to teaching LGBT health to baccalaureate nursing students in the United States was 2.12 hours.

**Primary Care Delivery in Ontario**

To understand how transgender care is delivered through primary care in Ontario, it is important to understand the organization of primary care models. Canada has a publicly funded, universal health insurance system which covers all medically necessary services (Laberge et al., 2016). Ontario uses a single payer insurance model which covers necessary medical services. All legal residents of Ontario are enrolled in the Ontario Health Insurance Plan (OHIP) and are required to provide their OHIP number to receive insured services (Rudoler et al., 2015). Primary care in Ontario has been reorganized over the last twenty years and now features multiple models of care delivery(Laberge et al., 2016). A model of care is a multidimensional concept describing the organization and delivery of healthcare services (Department of Health, 2007). In Ontario, the key variables are modes of physician payment, governance and support for interprofessional team practice. Common models include Fee-for-Service; Family Health Networks; Community Health Centers and Family Health Organizations (Laberge et al., 2016, Dahrouge et al., 2009, Health Force Ontario, 2017).

Fee-for-Service (FFS) model offers remuneration for each service provided as determined by the schedule of benefits (Dahrouge et al., 2009, Laberge et al., 2016). FFS practitioners may be solo or practice in small groups. Family Health Networks and Family Health Organizations are a blended-capitation model which requires physicians to work in groups of three or more (Glazier et al., 2012, Rudoler et al., 2015, Health Force Ontario, 2016). Family Health Organizations are also eligible to co-locate with a Family Health Team, (FHT). These teams receive additional funding for an interprofessional team which may include nurse practitioners, social workers, dietitians and pharmacists (Dahrouge et al., 2016, Laberge et al., 2016, Rudoler et al., 2015). Both Family Health Networks and Family Health Organizations involve contractual accountability to the Ministry of Health for the services provided within the blended capitation model. Lastly, a Community Health Center (CHC) is run by a community board to provide team-based primary care services to populations that have trouble securing services or “hard-to-serve” communities (Devlin et al., 2013). Health care practitioners in CHCs are salaried.

**Theoretical Framework**

The Normalization Process Theory (NPT) is an implementation theory and conceptual framework used to understand and explain the dynamic processes that occur during implementation of interventions in healthcare (May et al., 2011a). NPT is “concerned with the social organization of work (implementation), of making practices routine elements of everyday life (embedding), and of sustaining embedded practices in their social contexts (integration)” (May and Finch, 2009). NPT postulates that practice becomes routinely embedded or normalized as the result of people working to enact them (May et al., 2009).

NPT focuses on four theoretical constructs which describe mechanisms that are energized by the investments of the participants (May et al., 2011b). Coherence explores the means that a practice is made by a set of ideas that are socially defined and organized by competencies. These ideas and competencies hold the practice together (May and Finch, 2009). Cognitive participation of individuals in an organization promotes or inhibits the legitimation of the intervention, it is driven by the commitments of the participants (May and Finch, 2009, May et al., 2011b). The process of collective action is driven by the efforts of participants, it is the material and mental work that is done to enact a practice (May and Finch, 2009, May et al., 2011b). Collective action may be the reshaping of behaviours or actions or the reorganization of a collective purpose (May and Finch, 2009). The final theoretical construct is that patterns of collective action and outcomes are continuously evaluated through reflexive monitoring (May and Finch, 2009).

For this study the delivery and implementation of transgender primary care will be explored using NPT. Transgender primary care consists of any primary care services obtained by individuals who identify as transgender. This can include but is not limited to general episodic care, chronic care management, medical supervised transition, including providing access to and monitoring of transgender hormones, and counselling.

**Research Questions**

An exploratory case study will be used to answer the following research questions.

1. How is primary health care for transgender individuals delivered within different primary care models in Ontario?
2. What roles do different team members play in the delivery of primary care to transgender individuals?
3. How is the delivery of primary care for transgender individuals implemented within different primary care models in Ontario?
4. What has supported primary care practitioner’s capacity in developing their competence in delivering primary care to transgender individuals?
5. How does the implementation of primary care services for transgender individual compare across various models of primary care delivery?

**Methods**

**Research Design**

Qualitative methods explore, describe and explain phenomena (Creswell, 2007). This study will use an exploratory multiple case study design (Yin, 2014) to explore the implementation and delivery of primary health care for transgender individuals in Ontario. Case study research explores specific issues through examination of cases within a system (Creswell, 2007), and allows investigators to explore and retain a holistic and real world experience (Yin, 2014). The qualitative case study methodology allows for a phenomenon to be explored through different lenses by using a variety of data sources (Baxter and Jack, 2008).

As there are few documented examples of primary care services for transgender patients(Deutsch, 2011, Reisner et al., 2016, Esteva de Antonio et al., 2013, Wylie et al., 2016), a case study design allows for an in-depth exploration of how these services are implemented into practice and delivered in Ontario by exploring factors influencing this process. A multiple case study will allow for the exploration of the differences and similarities among the cases within different primary care models to develop a rich understanding of the phenomenon (Yin, 2014). Case study methodology is a valuable method for evaluating programs (Baxter and Jack, 2008). Yin’s case study methodology was selected as the qualitative design for this study as it allows for multiple case analysis and the use of both qualitative and quantitative data (Yin, 2014).

**Unit of Analysis – The Cases**

A purposeful sampling strategy will be used. Purposeful sampling involves selecting organizations which can purposefully inform the understanding of the research problem and central phenomenon being explored (Creswell, 2007). To explore and understand primary care for transgender individuals, three cases will be used for this research study. The three cases will represent different primary care delivery models in Ontario. The comparison of primary care programs and implementation across different primary care delivery models is vital in understanding the provision of care across the province. The cases will include one Solo FFS organization; one Community Health Center and one Family Health Team in Ontario. Primary care settings already providing primary care to transgender individuals will be included. The purpose for this is to understand the characteristics of the patient population, the different roles and preparation of practitioners to provide care to a transgender population, the process of implementation and factors which influence the program implementation.

**Participants**

Organizations currently providing primary care services to transgender individuals will be invited to participate in the study. Organizations will be identified as potential site through the researcher’s networks and Rainbow Health Ontario, a province-wide program to improve access to services and to promote the health of Ontario’s LGBT communities (Rainbow Health Ontario, 2018). Once organizational consent is obtained from the Director/ CEO, study participants will be recruited from each organization. Primary data will be collected from employees in each organization. Individuals will be eligible to participate in the study if they: 1) are currently involved in any way in the delivery of primary care services or have contact with transgender individuals; and 2) are fluent in English. The types of individuals invited to participate in interviews are listed in Table 1. The choice to include this array of primary care practitioners and clinical support staff (CSS) will help provide a rich and comprehensive descriptions of each case. As well, in Canada there is a trend for primary care to be provided by interdisciplinary teams. Participants who consent to participate will be contacted by the principal investigator to arrange an interview time, either in person or by telephone at the participant’s convenience. They will receive a $25 gift card to a coffee chain as a token of appreciation for their participation.

**Data Collection**

Data collection in case study research involves the use of multiple sources of evidence from within each case (Yin, 2014). The use of multiple sources of evidence allows a broader range of data and understanding of the cases (Yin, 2014). This study will collect data from a survey, semi-structured interviews, and documentary evidence.

All participants, except CSS will be asked to complete the NOrmalization MeAsure Development (NoMAD) tool (Finch et al., 2015), a 23-item instrument based on the constructs of NPT for measuring implementation processes from the viewpoint of professionals involved in the health care program or intervention implementation (Finch et al., 2013). The development methods of the NoMAD tool included item generation, workshops, interviews, item quality appraisal, and theory validation (Finch et al., 2013). Psychometrics for the NoMAD instrument have been determined and will be reported in a forthcoming publication. T. Finch, from the Northumbria University and the lead author of the publication have stated that "The NoMAD instrument has good face validity, construct validity and internal consistency, for assessing staff perceptions of factors relevant to embedding interventions that change their work practices" (personal communication, May 24, 2017). The tool developers recommend that the tool be adapted for specific use, suggesting replacing the word “intervention” with term that would be most familiar to study participants (Normalization Process Theory, 2017). For this study, “intervention” was replaced with “primary care for transgender patients”. Participants will complete the NoMAD survey anonymously and place the completed forms in a sealed drop box located at their organization prior to their interview.

Semi-structured interviews will be conducted by the principal investigator to collect qualitative data that addresses the research questions 1 to 4. The use of semi-structured interviews allows for the researcher to ask specific questions related to the study purpose and still allow for the participant to freely express their opinion and thoughts about the topic (Burns and Grove, 2005, Speziale and Carpenter, 2007). Participants will be interviewed using the developed interview guides. Questions asked to participants are listed in Table 2. The use of open-ended questions allows participants to contribute as much detail as they see fit and allow the researcher to ask follow up questions to probe further (Creswell, 2007, Turner, 2010). Areas to be explored in the interviews include practitioners and CSSs experience with transgender patients, the development and implementation of the primary care program, program demographics and the role of the members of the team.

A pilot test will be done of the interview guide. The purpose of the pilot test will be to determine if there are any flaws, limitations or weaknesses in the interview design (Turner, 2010). The pilot test will be done at the first case study site as part of data collection. Participants will be interviewed using the interview guide and asked in the consent if they agree to be contacted again for further questions should gaps be identified in the interview guide. If necessary, revisions will be made to the interview guide.

Primary care practitioner’s interviews will take approximately 90 minutes to complete. Interviews will CSS will take approximately 45 minutes. Consent for recording will be obtained and interviews will be transcribed verbatim. Participants will be given a copy of the interview guide before the interview so that they can reflect on the questions and develop detailed responses and consider anonymous case examples to discuss during the interview. Field notes will also be used by the researcher to document observations, consider the context of the setting, potential themes, insights, and further issues to explore which arise within the interview. Participants will be asked to complete a demographic questionnaire.

Documentary evidence, including administrative reports, proposals, evaluations, and other relevant internal records, will also be gathered from each case. Use of documentary evidence in case study research can provide specific insight into the development and implementation of the delivery of primary care for transgender patients at each organization. However, a weakness with using documentary evidence is that it can be difficult to retrieve and not the same at each organization (Yin, 2014).

**Data Analysis**

Qualitative data analysis will be conducted concurrently with data collection to allow for clarification and further exploration of emerging concepts and themes in future interviews (Baxter and Jack, 2008, Miles et al., 2014). Yin (2014) recommends starting the data analysis process by exploring the data, looking for patterns, insights or concepts. All qualitative data will be transcribed and reviewed for accuracy. NVivo, a qualitative data management and analysis software will be used for data analysis (QSR International, 2017). Deductive codes will be initially developed from the concepts of NPT, the research purpose and research questions. Following this, inductive coding will be done as new codes emerge during the data collection (Miles et al., 2014). Line by line coding of all data sources will follow within the first cycle coding (Miles et al., 2014). Second level coding will be completed by grouping first cycle data into smaller categories, themes or constructs (Miles et al., 2014) within the large concepts from NPT. Codes that do not fit under NPT concepts will be included. The code book will be reviewed with the research team to obtain agreement on the organization of codes and naming of themes.

Qualitative content analysis will be used to summarize the informational content of the data (Sandelowski, 2000) and is a systematic and objective means of describing and quantifying phenomena (Elo and Kyngäs, 2008). An inductive approach will be used as there is not enough former knowledge about the phenomenon (Elo and Kyngäs, 2008). This approach moves from the specific to the general, allowing for particular instances to be observed and then combined into a general statement (Elo and Kyngäs, 2008).

Further data analysis will be done using cross-case synthesis (Yin, 2014, Baxter and Jack, 2008). Cross-case synthesis is a method of data analysis which facilitates the comparison of commonalities and differences within the cases and creates word tables to display the data from individual cases to form categories (Yin, 2014, Khan and Vanwynsberghe, 2008). NVivo queries will be used to create matrices to support cross-case synthesis which can identify differences and commonalties (Miles et al., 2014, Yin, 2014).

Quantitative data from the NoMAD instrument will be analyzed using SPSS, however there is currently no existing literature on how the analysis of data collected with the NoMAD instrument should be analyze (Finch et al., 2015). The NoMAD instrument is divided into Option A and Option B. For analysis and interpretation, the Likert response format in Option A will be recoded to 1 = *strongly disagree* to 5 = *strongly agree.* Descriptive statistics using means and standard deviation will be used to described participants responses to Option A, by NPT construct and each individual question (Normalization Process Theory, 2017). Frequencies and percentages will be used to describe responses to Option B. Mean NPT construct scores will be compared across the three different primary care models. Independent t-tests will be conducted to determine if there are any statistically significant differences between the primary care models using a significance level of 0.05.

Convergence of qualitative and quantitative data will be completed (Baxter and Jack, 2008, Yin, 2014)to given any overall understanding of how primary care services are implemented and delivered in Ontario.

**Validity and Reliability**

Yin (2014) identifies four tests for judging the quality of case study research designs: construct validity, internal validity, external validity, and reliability. The following methodological considerations and strategies will address the validity and reliability across the overall study. Use of replication logic in multiple case studies will ensure external validity. Literal replication logic states that each case must be carefully selected to predict similar results (Yin, 2014). If cases are contradictory, initial questions are revised and retested with another case to ensure external validity (Yin, 2014). To confirm construct validity multiple sources of evidence will be collected and a chain of evidence will be established (Yin, 2014). Analytical techniques during data analysis such as pattern matching, explanation building and addressing rival explanations will be done to ensure internal validity (Yin, 2014).

Lincoln and Guba (1985) identify four strategies to ensure trustworthiness and reliability in qualitative research which include credibility, transferability, dependability and confirmability. These four strategies will be used in this study to ensure trustworthiness of results. Multiple methods of triangulation will be used to ensure credibility, dependability and confirmability. Data methods triangulation will be done by collecting and comparing data from multiple sources (Krefting, 1991, Patton, 1999) which include individual interviews, a survey, and documentary evidence. Triangulation of data sources will be done by using three different cases to collect data to maximize the range of data (Patton, 1999, Krefting, 1991). Investigator triangulation will occur with the use of the research team to support the analysis and interpretation of the results (Krefting, 1991, Patton, 1999).

Credibility will enhanced with individual interviews being guided by the use of a consistent semi-structured interview guide (Krefting, 1991). A field journal for researcher self-reflection and ideas will but maintained as a method to ensure credibility and confirmability (Guba, 1981). The use of the journal will allow for reflection on potential biases and preconceived assumptions to be addressed (Krefting, 1991). Transferability will be established though a thick description, which is a detailed account of the field experience (Lincoln and Guba, 1985, Guba, 1981). This thick description of context will allow for readers to evaluate the extent to which the conclusions drawn are transferable to other times, settings, situations, and individuals (Guba, 1981).

**Ethical Considerations**

This study was approved by the Research Ethics Board at McMaster University. All participation in this study will be voluntary. Organizational consent from each case will be obtained, as well as informed consent from all participants (Burns and Grove, 2005). All data will remain confidential and identifying information will be removed (Burns and Grove, 2005). There is a small possibility that due to the specialization of the organizations providing transgender services they may be recognizable to some local readers of the study findings. This will be discussed with the directors of these organizations as well as all participants in the consent and prior to any released reports. The potential benefits of participating in the study will be outlined to the participants, including the use of information from this study to develop and improve the provision of primary health care to transgender individuals.

**Limitations**

Several limitations need to be considered. This study will explore three cases; therefore, results may not be transferrable to organizations in different contexts. However, a thick description of each case, will permit readers to determine the relevance of the results to their setting. Similarly, this study will only explore three primary care models, thus results may not be transferable to other primary care models. The delivery and implementation of transgender care will only be explored retrospectively at one point in time which limits the understanding of implementation over time and has the potential for recall bias. Further, practitioners who may have been instrumental in the implementation may have left the organization.

**Declarations**

**Acknowledgements –** Not Applicable

**Funding –** This work is supported by the Dorothy C. Hall Chair in Primary Health Care in Nursing and the Transdisciplinary Understanding and Training on Research Primary Health Care trainee award.

**Conflict of Interest -** None

**Ethics approval and consent to participate -** This study proposal received ethics approval from the Research Ethics Board at McMaster University project #3751. All participation in this study is voluntary. Written consent for all participants will be obtained.

References

ALEGRIA, C. A. 2011. Transgender identity and health care: implications for psychosocial and physical evaluation. *Journal of the American Academy of Nurse Practitioners,* 23**,** 175-82.

BAUER, G. R., HAMMOND, R., TRAVERS, R., KAAY, M., HOHENADEL, K. M. & BOYCE, M. 2009. "I don't think this is theoretical; this is our lives": how erasure impacts health care for transgender people. *Journal of the Association of Nurses in AIDS Care,* 20**,** 348-61.

BAXTER, P. & JACK, S. 2008. Qualitative case study methodology: Study design and implementation for novice researchers. *The Qualitative Report,* 13**,** 544-559.

BURNS, N. & GROVE, S. 2005. *The practice of nursing research: Conduct, critique, and utilization,* St. Louis, MO, Elsevier Saunders.

COLEMAN, E., BOCKTING, W., BOTZER, M., COHEN-KETTENIS, P., DECUYPERE, G., FELDMAN, J., FRASER, L., J, G., KNUDSON, G., MEYER, W., MONSTREY, S., ALDER, R., BROWN, G., DEVOR, A., EHRBAR, R., ETTNER, R., EYLER, E., GAROFALO, R., KARASIC, D., LEV, A., MAYER, G., MEYER-BAHLBURG, H., HALL, B., PFAEFFLIN, F., RACHLIN, K., ROBINSON, B., SCHECHTER, L., TANGPRICHA, V., VAN TROTSENBURG, M., VITALE, A., WINTER, S., WHITTLE, S., WYLIE, K. & ZUCKER, K. 2012. Standards of care for the health of transsexual, transgender, and gender-non conforming people, version 7. *International Journal of Transgenderism* 13**,** 165-232.

CRESWELL, J. 2007. *Qualitative inquiry & research design: Choosing among five approaches,* Thousand Oaks, CA, Sage Publications, Inc. .

CRUZ, T. M. 2014. Assessing access to care for transgender and gender nonconforming people: a consideration of diversity in combating discrimination. *Social Science & Medicine,* 110**,** 65-73.

DAHROUGE, S., HOGG, W., RUSSELL, G., GENEAU, R., KRISTJANSSON, E., MULDOON, L. & JOHNSTON, S. 2009. The Comparison of Models of Primary Care in Ontario (COMP-PC) study: methodology of a multifaceted cross-sectional practice-based study. *Open Medicine,* 3.

DAHROUGE, S., HOGG, W., YOUNGER, J., MUGGAH, E., RUSSELL, G. & GLAZIER, R. 2016. Primary Care Physician Panel Size and Quality of Care: A Population-Based Study in Ontario, Canada. *The Annals of Family Medicine,* 14**,** 26-33.

DEPARTMENT OF HEALTH, S. W. A. 2007. Model of care: Overview and guideline State of Western Australia

DEUTSCH, M. 2011. Use of the informed consent model in the provision of cross-sex hormone therapy: A survery of the practices of selected clinics. *International Journal of Transgenderism,* 13**,** 140-146.

DEVLIN, R. A., HOGG, W., ZHONG, J., SHORTT, M., DAHROUGE, S. & RUSSELL, G. 2013. Practice size, financial sharing and quality of care. *BMC Health Services Research,* 13**,** 446.

ELO, S. & KYNGÄS, H. 2008. The qualitative content analysis procedure. *Journal of Advanced Nursing,* 62**,** 107-115.

ESTEVA DE ANTONIO, I., GOMEZ-GIL, E. & GIDSEEN GROUP 2013. Coordination of healthcare for transsexual persons: a multidisciplinary approach. *Current Opinion in Endocrinology, Diabetes & Obesity,* 20**,** 585-91.

FINCH, T., GIRLING, M., MAY, C., MAIR, F., MURRAY, E., TREWEEK, S., STEEN, I., MCCOLL, E., DICKINSON, C. & RAPLEY, T. 2015. *NoMAD: Implementation measure based on Normalization Process Theory. [Measurement instrument].* [Online]. Available: http://www.normalizationprocess.org. [Accessed May 6, 2017 2017].

FINCH, T. L., RAPLEY, T., GIRLING, M., MAIR, F. S., MURRAY, E., TREWEEK, S., MCCOLL, E., STEEN, I. N. & MAY, C. R. 2013. Improving the normalization of complex interventions: Measure development based on normalization process theory (NoMAD). *Implementation Science,* 8**,** 43.

FLORES, A., HERMAN, J., GATES, G. & BROWN, T. 2016. How many adults identify as transgender in the United States? : UCLA - The Williams Institute

GARDNER, I. H. & SAFER, J. D. 2013. Progress on the road to better medical care for transgender patients. *Current Opinion in Endocrinology, Diabetes & Obesity,* 20**,** 553-8.

GIBLON, R. & BAUER, G. 2017. Health care availability, quality, and unmet need: A comparison of transgender and cisgender residents in Ontario, Canada. *BMC Health Services Research,* 17.

GLAZIER, R., KOOP, A., SCHULTZ, S., KIRAN, T. & HENRY, D. 2012. All the right interventions but few of the desired results: Lessons on access to primary care from Ontario's patient enrolment models *Healthcare Quarterly,* 15**,** 17-21.

GUBA, E. 1981. ERIC/ECTJ Annual review paper: Criteria for assessing the trustworthiness of naturalistic inquiries. *Educational Communication and Technology,* 29**,** 75-91.

HEALTH FORCE ONTARIO. 2016. *Family Practice Models* [Online]. Available: www.healthforceontario.ca/en/Home/Physicins/Training_1_Practising_in_Ontario/Physicians_Roles/Family_Practice_Models#ccm [Accessed December 16, 2016 2016].

HEALTH FORCE ONTARIO. 2017. *Family Practice Compensation Models* [Online]. Available: http://www.healthforceontario.ca/en/Home/Physicians/Training_%7C_Practising_in_Ontario/Physician_Roles/Family_Practice_Models/Family_Practice_Compensation_Models [Accessed May 5, 2017 2017].

HEINZ, M. & MACFARLANE, D. 2013. Island Lives: A Trans community needs assessment for Vancouver Island *SAGE Open,* 3**,** 2158244013503836.

INSTITUTE OF MEDICINE 2011. *The Health of Lesbian, Gay, Bisexual, and Transgender People: Building a Foundation for Better Understanding* Washington, DC, National Academies Press

KHAN, S. & VANWYNSBERGHE, R. 2008. Cultivating the under-minded: Cross case analysis of knowledge mobilization. *Forum: Qualitative Social Research,* 9.

KREFTING, L. 1991. Rigor in qualitative research: The assessment of trustworthiness. *The American Journal of Occupational Therapy* 45**,** 214-222.

LABERGE, M., WODCHIS, W., BARNSLEY, J. & LAPORTE, A. 2016. Efficiency of Ontario primary care physicians across payment models: A stochastic frontier analysis. *Health Economics Review,* 6.

LIM, F., JOHNSON, M. & ELIASON, M. 2015. A national survey of faculty knowledge, experience, and readiness for teaching lesbian, gay, bisexual, and transgender health in baccalaureate nursing programs. . *Nursing Education Perspectives,* May/June.

LINCOLN, Y. & GUBA, E. 1985. *Naturalistic inquiry,* Newbury Park, CA, Sage Publications.

MAKADON, H. J. 2011. Ending LGBT invisibility in health care: the first step in ensuring equitable care. *Cleveland Clinic Journal of Medicine,* 78**,** 220-4.

MAY, C. & FINCH, T. 2009. Implementing, embedding, and integrating practices: An outline of Normalization Process Theory. *Sociology* 43**,** 535-554.

MAY, C., FINCH, T. & BALLINI, L. 2011a. Evaluating complex interventions and health technologies using normalization process theory: development of a simplified approach and web-enabled toolkit. *BMC Health Serv Res,* 11.

MAY, C., FINCH, T., BALLINI, L., MACFARLANE, A., MAIR, F., MURRAY, E., TREWEEK, S. & RAPLEY, T. 2011b. Evaluating complex interventions and health technologies using normalization process theory: development of a simplified approach and web-enabled toolkit. *BMC health services research,* 11**,** 245-255.

MAY, C., MAIR, F., FINCH, T., MACFARLANE, A., DOWRICK, C., TREWEEK, S., RAPLEY, T., BALLINI, L., ONG, B., ROGERS, A., MURRAY, E., ELWYN, G., LEGARE, F., GUNN, J. & MONTORI, V. 2009. Development of a theory of implementation and integration: Normalization Process Theory. *Implementation Science,* 4.

MILES, M., HUBERMAN, M. & SALDANA, J. 2014. *Qualitative data analysis: A methods sourcebook,* Washington, DC, Sage.

NORMALIZATION PROCESS THEORY. 2017. *How to use NoMAD?* [Online]. Available: http://www.normalizationprocess.org/nomad-study/ [Accessed May 30, 2017].

OBEDIN-MALIVER, J., GOLDSMITH, E. S., STEWART, L., WHITE, W., TRAN, E., BRENMAN, S., WELLS, M., FETTERMAN, D. M., GARCIA, G. & LUNN, M. R. 2011. Lesbian, gay, bisexual, and transgender-related content in undergraduate medical education. *JAMA,* 306**,** 971-7.

PATTON, M. 1999. Enhancing the quality and credibility of qualitative analysis. *Health Services Research,* 34**,** 1189–1208.

QSR INTERNATIONAL. 2017. *Nvivo 11* [Online]. Available: http://www.qsrinternational.com/nvivo-product [Accessed March 24, 2017].

RAINBOW HEALTH ONTARIO 2015. *Guidelines and protocols for hormone therapy and priary healthcare for trans clients* Toronto, ON, Bourne, A. .

RAINBOW HEALTH ONTARIO. 2018. *Rainbow Health Ontario a Program of Sherbourne Health* [Online]. Available: https://www.rainbowhealthontario.ca/ [Accessed].

REISNER, S., BRADFORD, J., HOPWOOD, R., GONZALEZ, A., MAKADON, H., TODISCO, D., CAVANAUGH, T., VANDERWARKER, R., GRASSO, C., ZASLOW, S., BOSWELL, S. & MAYER, K. 2015. Comprehensive transgender healthcare: the gender affirming clinical and public health model of Fenway Health. *Journal of Urban Health,* 92**,** 584-92.

REISNER, S., RADIX, A. & DEUTSCH, M. 2016. Integrated and gender-affirming transgender clinical care and reserach *Journal of Acquired Immune Deficiency Syndromes,* 72**,** S235-S242.

ROBERTS, T. K. & FANTZ, C. R. 2014. Barriers to quality health care for the transgender population. *Clinical Biochemistry,* 47**,** 983-7.

RUDOLER, D., DEBER, R., BARNSLEY, J., GLAZIER, R. H., DASS, A. R. & LAPORTE, A. 2015. Paying for Primary Care: The Factors Associated with Physician Self‐selection into Payment Models. *Health Economics,* 24**,** 1229-1242.

SANCHEZ, N. F., SANCHEZ, J. P. & DANOFF, A. 2009. Health care utilization, barriers to care, and hormone usage among male-to-female transgender persons in New York City. *American Journal of Public Health,* 99**,** 713-9.

SANDELOWSKI, M. 2000. Whatever happened to qualitative description? . *Reserach in Nursing & Health* 23**,** 334-340.

SNELGROVE, J. W., JASUDAVISIUS, A. M., ROWE, B. W., HEAD, E. M. & BAUER, G. R. 2012. "Completely out-at-sea" with "two-gender medicine": a qualitative analysis of physician-side barriers to providing healthcare for transgender patients. *BMC Health Services Research,* 12**,** 110.

SPEZIALE, H. & CARPENTER, D. 2007. *Qualitative research in nursing* Philadelphia, PA, Lippincott Williams & Wilkins.

TURNER, D. 2010. Qualitative interview design: A practical guide for novice investigators. *The Qualitative Report,* 15**,** 754-760.

WHITE, W., BRENMAN, S., PARADIS, E., GOLDSMITH, E. S., LUNN, M. R., OBEDIN-MALIVER, J., STEWART, L., TRAN, E., WELLS, M., CHAMBERLAIN, L. J., FETTERMAN, D. M. & GARCIA, G. 2015. Lesbian, Gay, Bisexual, and Transgender Patient Care: Medical Students' Preparedness and Comfort. *Teaching and Learning in Medicine,* 27**,** 254-263.

WYLIE, K., KNUDSON, G., KHAN, S. I., BONIERBALE, M., WATANYUSAKUL, S. & BARAL, S. 2016. Serving transgender people: clinical care considerations and service delivery models in transgender health. *The Lancet,* 388**,** 401-411.

YIN, R. 2014. *Case study reserach: Design and methods,* Thousand Oaks, CA, Sage Publications, Inc.
